# Kagome Hollow Core Fiber-Based Mid-Infrared Dispersion Spectroscopy of Methane at Sub-ppm Levels

**DOI:** 10.3390/s19153352

**Published:** 2019-07-31

**Authors:** Karol Krzempek, Krzysztof Abramski, Michal Nikodem

**Affiliations:** 1 Laser & Fiber Electronics Group, Faculty of Electronics, Wroclaw University of Science and Technology, Wybrzeze Wyspianskiego 27, 50-370 Wroclaw, Poland; 2 Department of Optics and Photonics, Faculty of Fundamental Problems of Technology, Wroclaw University of Science and Technology, 50-370 Wroclaw, Poland

**Keywords:** laser spectroscopy, gas detection, hollow-core fiber, kagome fiber, anti-resonant fiber, methane, dispersion spectroscopy

## Abstract

In this paper, we demonstrate the laser-based gas sensing of methane near 3.3 µm inside hollow-core photonic crystal fibers. We exploit a novel anti-resonant Kagome-type hollow-core fiber with a large core diameter (more than 100 µm) which results in gas filling times of less than 10 s for 1.3-m-long fibers. Using a difference frequency generation source and chirped laser dispersion spectroscopy technique, methane sensing with sub-parts-per-million by volume detection limit is performed. The detection of ambient methane is also demonstrated. The presented results indicate the feasibility of using a hollow-core fiber for increasing the path-length and improving the sensitivity of the mid-infrared gas sensors.

## 1. Introduction

Infrared laser spectroscopy is a sensitive and selective technology with a number of practical applications. The simplest and frequently used solution for further improvement of the minimum detection limit (MDL) is to increase the sensing path-length using multi-pass cells. This approach typically leads to sensitivities at parts-per-million or parts-per-billion by volume levels (ppmv and ppbv, respectively) [1,2,3,4,5]. Unfortunately, multi-pass cells have drawbacks. Long optical paths usually require either relatively large volumes or a substantial number of passes which results in poor throughput of the cell. Moreover, multi-pass cells require precise optical alignment and are often sources of drifts (due to optomechanical instabilities) [6]. Hollow-core fiber (HCF) is an interesting alternative to multi-pass cells. HCF can be used as a long gas cell that is mechanically stable, easier to align, and provides exceptional path-length-to-volume ratios. Examples of laser spectroscopy using long capillary with reflective inner coating were presented in [7,8]. Unfortunately, when this type of fiber is used for gas sensing, multimode propagation or scattering on inner surfaces may occur, possibly leading to the presence of optical fringes which strongly limit the performance when standard absorption-based methods (such as tunable diode laser absorption spectroscopy or wavelength modulation spectroscopy) are used. Similar limitations are observed in the configuration incorporating photonic bandgap (PBG) photonic crystal fibers (PCFs) [9,10]. In these systems, achieving reasonable performance usually requires using more sophisticated sensing techniques [11,12,13]. Another drawback of the PBG-HCFs is that they typically have small core diameters (below 20 µm, with some exceptions [14]) which makes filling the fiber with the gas sample difficult. 

In this paper, we present high-resolution laser spectroscopy in the mid-infrared (mid-IR) region using Kagome-type negative curvature hollow-core PCF. This type of fiber has several advantages over PBG-PCFs. Its most important properties are reduced coupling between the cladding modes and the core modes and large core diameter. Using Kagome-type HC-PCF designed for mid-infrared wavelengths, an all-fiber difference frequency generation (DFG) source [15,16] and chirped laser dispersion spectroscopy (CLaDS) technique [17,18], methane (CH_4_) detection at 3.33 µm with sub-ppmv×m sensitivity is demonstrated. The obtained performance is almost an order of magnitude better (the improvement factor was approximately 8.22) comparing to the results obtained using the wavelength modulation spectroscopy technique [19], and it indicates great potential for compact and highly sensitive mid-infrared sensors that rely on HCFs and molecular dispersion spectroscopy.

## 2. Experimental Setup

**Figure 1:**
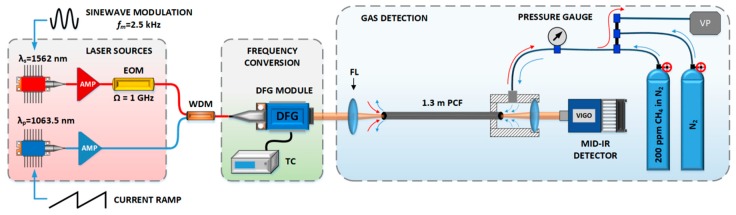
Schematic diagram of the experimental setup. AMP—fiber amplifier, WDM—wavelength division multiplexer, DFG—difference-frequency generation, TC—temperature controller, FL—focusing lens, PCF—photonic crystal fiber, VP—vacuum pump.

The experimental arrangement of the sensor setup is schematically shown in Figure 1.

**Figure 2:**
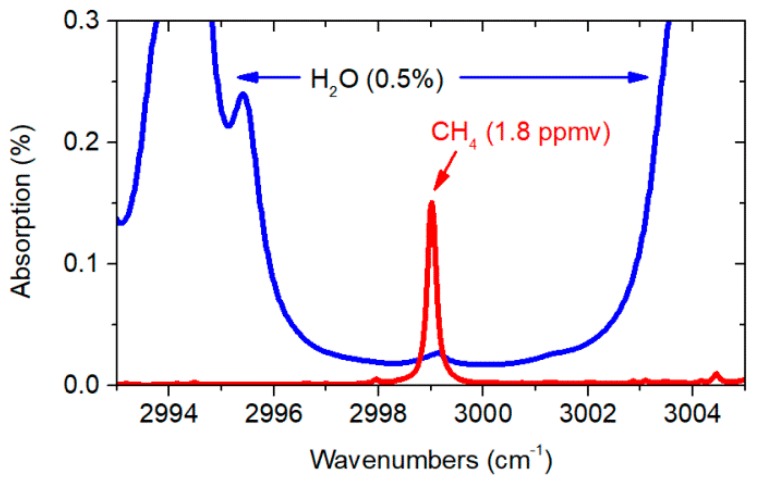
Absorption spectra of methane and water vapor retrieved from the HITRAN database [20]. This simulation is for 1.3 m path-length and pressure of 760 Torr.

Coherent mid-infrared light was obtained through the difference frequency generation (DFG) process in a periodically poled lithium niobate (PPLN) crystal. Two distributed feedback (DFB) laser diodes with center wavelengths at 1063.5 nm and 1562 nm were used as seed sources for the DFG module. They were driven, and their temperatures were stabilized using commercially available controllers (Thorlabs, model CLD1015). Light from the seed sources was amplified up to 90 mW (at 1562 nm) and 80 mW (at 1063.5 nm) using standard erbium-doped and ytterbium-doped fiber amplifiers. Both wavelengths were combined using a fiber coupler. For optimum quasi-phase-matching, the DFG module was temperature-stabilized at 60 °C. With a highly efficient fiber-pigtailed DFG module, comprising a 40 mm-long PPLN waveguide crystal with a very high conversion efficiency (21%/W) [15], the nonlinear optical mixing process resulted in generating approximately 1 mW of mid-infrared light centered at ~3.33 µm. This wavelength enables targeting P(2) transition in the v3 band of methane, isolated from water vapor absorption lines, as shown in Figure 2 (proper selection of the target transition is necessary to avoid interference from the other species and guarantees high selectivity). A 1.3 m-long piece of Kagome-type HC-PCF (commercially available from GLOphotonics) was used as a gas cell in this experiment. Manufactured from silica glass, the HCF had two broad transmission bands, first between 2 µm and 2.4 µm, and second between 2.8 and 3.5 µm. The core diameter was ~116 µm. The mid-IR beam was coupled into HC-PCF using an f = 75 mm lens. Filling the fiber with gas samples was possible through a custom-made air-tight housing, encapsulating the outlet of the fiber. 

For filling the HCF with gas from a cylinder, an overpressure of ~850 Torr was used to ensure constant flow through the fiber. For filling with ambient air, a vacuum pump was used. The pressure inside the air-tight housing was reduced down to ~500 to ~600 Torr so that ambient air could be sucked through the front of the HCF. 

The flow rate was estimated to be between 100 and 320 µL/min. The beam exiting the fiber was focused onto a fast mercury-cadmium-telluride (MCT) detector (VIGO System, model PVI-4TE-8-0.5×0.5).

The detection technique implemented here was chirped laser dispersion spectroscopy, CLaDS. In contrast to typically used methods which target molecular absorption, CLaDS relies on measuring changes of the real part of the refractive index in the vicinity of absorption line [17,18,21]. In order to perform CLaDS measurements, a high-speed electro-optical intensity modulator was included in the setup to modulate the ~1.56 µm light, creating two sidebands separated from the carrier by the modulation frequency Ω = 1 GHz (limited by the bandwidth of the photodetector). After the DFG process, these three waves were duplicated at 3.33 µm. They were subsequently sent through the sample and focused onto a detector where heterodyne beat note at Ω was created. Its phase depends on the phases of the optical waves which are modified by the refractive index changes in the vicinity of methane transition (i.e., molecular dispersion). In our setup, sinusoidal modulation of the wavelength of the laser diode at ~1.56 µm was used to translate the phase of the beat note into changes of its frequency (as described in details in [17]). The modulation frequency *f*_m_ = 2.5 kHz was used, and variations of the frequency of the beat note were retrieved through the frequency demodulation at Ω and subsequent digital filtering of 2 × *f*_m_ component [22]. The demodulation was performed using an radio frequency (RF) spectrum analyzer, Rohde & Schwarz, model FSV7, with a demodulation bandwidth set to 12.5 kHz. The CLaDS spectroscopic information is encoded into the frequency of the beat note; therefore, this technique is highly immune to fluctuations of the optical power in the system. It also provides a linear response for a vast range of concentrations and generates signals without baseline. These features make CLaDS particularly useful for the DFG- and HCF-based system presented here. 

## 3. Results

### 3.1. System Characterization

**Figure 3:**
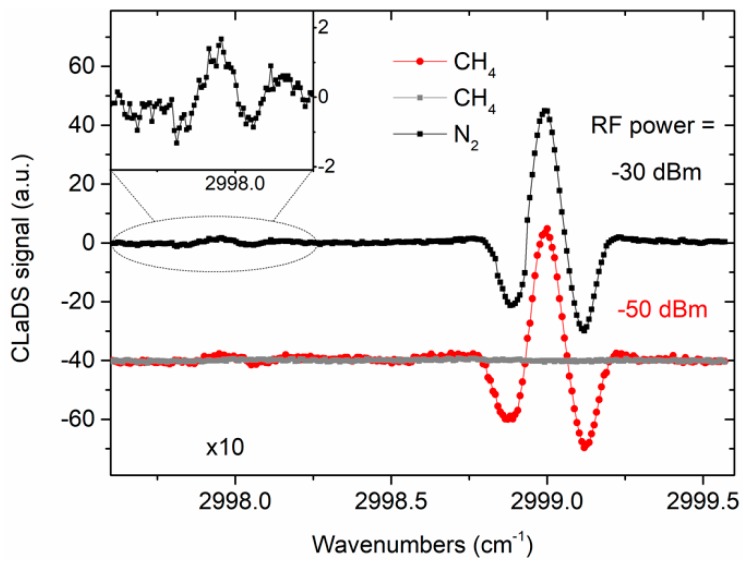
Spectral scans recorded when the HCF was filled with methane (black and red) and nitrogen (gray). Red and gray plots were vertically shifted by 40 a.u. for viewing purposes. Two methane signals were acquired at different powers of the beat note (−30 and −50 dBm) to show immunity of CLaDS signal amplitude to power variations. The inset shows another methane line near 2998 cm^−1^. Note that the same arbitrary units (a.u.) are used in other figures in this paper.

Figure 3 shows three spectral scans: two recorded after filling the fiber with a calibrated mixture of 200 ppmv (+/−3%) CH_4_ in nitrogen and the third measured with pure nitrogen. The wavelength modulation amplitude of ~5.8 GHz was determined experimentally to provide a maximal CLaDS signal amplitude (this was obtained through modulation of the laser current with a peak-to-peak amplitude of 15 mA). To enable recording the full CLaDS spectrum of the target transition, input light from the ~1 µm laser was additionally current-ramped at 25 mHz. A single data point was acquired within 100 ms. With an additional 60 ms needed for data processing, the resulting data acquisition rate was 6.25 Hz (250 points/spectrum). The spectra of methane were recorded for two different RF beat note powers at the input of the demodulator (RF power was reduced through misalignment of the detector). This results in a small increase of noise but has no effect on the signal amplitude, which remained unchanged. There is also no baseline in the measured signals. 

In Figure 3, part of the spectra was zoomed to show another methane transition near 2998 cm^−1^. According to HITRAN, this line (also visible in the simulation presented in Figure 2) is ~35 times weaker than the transition at 2999 cm^−1^ which is in good agreement with presented measurements.

**Figure 4:**
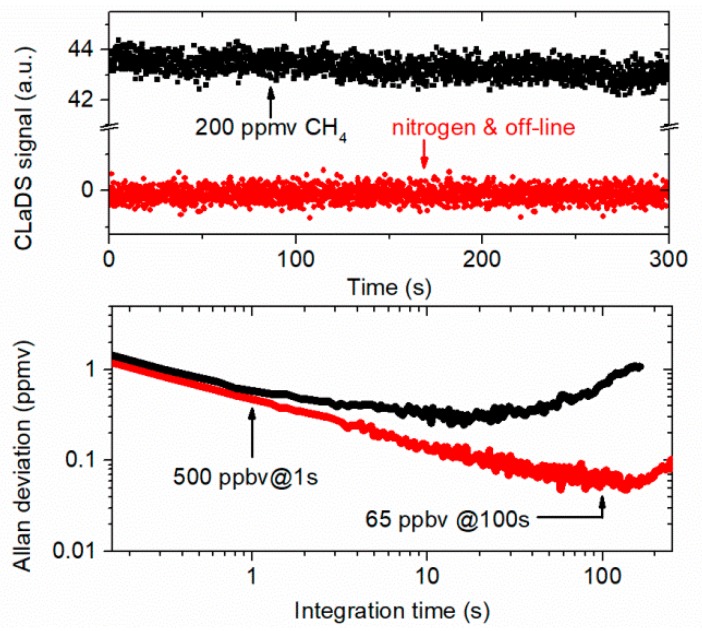
Top: Long-term CLaDS signal measurements performed at the center of the transition at 2999 cm^−1^ for 200 ppmv of methane and at 2999.5 cm^−1^ for nitrogen. Bottom: Allan deviation analysis for both cases.

Thanks to the baseline-free nature of the CLaDS, continuous concentration monitoring could be performed without full spectral scanning. Instead, the frequency of the mid-infrared radiation could be adjusted to the peak of the CLaDS signal and only the signal amplitude could be measured (this amplitude is proportional to the molecular concentration [22]). Figure 4 shows recorded signal amplitudes when HCF was filled with methane (200 ppmv) and pure nitrogen. For measurement with pure nitrogen, the DFG wavelength was additionally tuned by ~0.5 cm^−1^ in order to avoid signal from ambient methane within the free-space section of the setup. These two signals were used to calculate the Allan Deviation (Allan–Werle plot is presented in Figure 4) which can be used to estimate the minimum detectable CH_4_ concentration of the system [23]. For short integration times, the extracted signal is dominated by white noise. Sensitivity at 1 s is ~500 ppbv which corresponds to a minimum fractional absorption of 4 × 10^−4^. In the case of 200 ppmv CH_4_ measurement, no improvement is present for the integration times longer than a few seconds. The observed drift is probably due to the instability of the wavelength of the two near-infrared laser diodes (no active line-locking was used, we have only stabilized the temperature of two near-infrared laser diodes using the temperature controllers mentioned earlier). This was confirmed with zero-gas analysis for which white noise-like performance is observed up to ~100 s (65 ppbv corresponds to a fractional absorption of 5.2 × 10^−5^). Allan deviation analysis indicates good stability of the setup. However, increasing the integration time above 200 s does not result in further improvement of the detection limit. This may be due to the presence of optical fringes in the system (which might be generated by the interference of spatial modes inside HCF or by unwanted reflections inside the DFG section of the setup) or some thermal drifts in the setup.

### 3.2. System Demonstration

**Figure 5:**
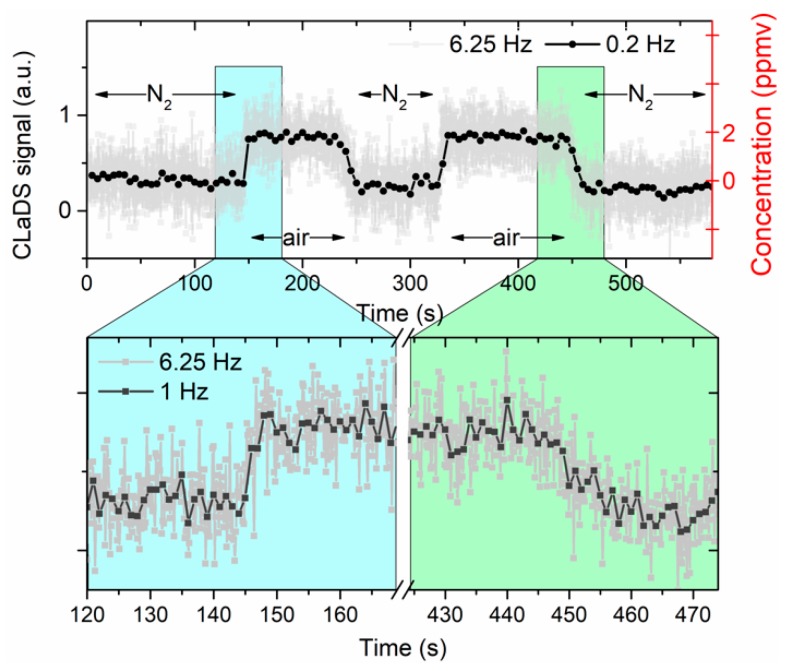
Retrieved CLaDS signal when HCF was alternately flushed with nitrogen and ambient air. Data points were acquired at 6.25 Hz (0.2 Hz and 1 Hz are the time series after averaging). Right axis shows methane concentration inside the hollow-core fiber, calculated using the two-point calibration shown in Figure 4.

For system demonstration, HCF was alternately filled with nitrogen and laboratory air (ambient concentration of methane is typically ~1.8 ppmv [24]). The recorded signal is presented in Figure 5 (signal measured during gas switching is shown in more details). The gas exchange time was found to be between 5 and 15 s. Slightly shorter time was obtained when HCF was being filled with ambient air. This is due to a larger pressure difference between the inlet and outlet immediately after the vacuum pump was switched on. A non-zero signal amplitude for N_2_ measurement is (primarily) from ambient methane measured within the free-space section of the setup (approximately 60 cm). the standard deviation of the recorded data points is below 300 ppbv (for time series with 1 s averaging) which is consistent with detection limit estimated based on the Allan–Werle analysis. 

## 4. Discussion

In this paper, a setup for laser-based methane sensing inside Kagome-type hollow-core fiber was presented. Previous work on HCF-based methane detection reported in the literature were mainly focused on PBG-type fibers. In [25], the setup for methane detection at 1670 nm was demonstrated. Using methane concentrations between 750 ppmv and 18750 ppmv, the detection limit of 10 ppmv using 5.1-m-long fiber was predicted. However, the presented spectral scans show relatively large fringes. Moreover, the diffusion time for the gas along this fiber was very long (~7 min) [26]. Another example of methane sensing inside the PBG-HCF in the near-infrared region is shown in [9]. Even though the presented experiments were conducted using large concentrations of methane (5%), also in this case, strong optical fringes are visible and they significantly limit the achievable sensitivity. The diffusion time in this case is even longer (20 min). In [27], methane detection beyond 3 µm was demonstrated with the PBG-type HCF, optical parametric oscillator (OPO) source and Fourier Transfer Spectrometer. The authors predicted that the sensitivity down to 50 ppmv could be achieved with this approach (for 3-m-long fibers). More recently, in [28], another example of methane sensing beyond 3 µm inside the PBG-HCF was shown. Using a mid-infrared supercontinuum source and an optical spectrum analyzer, a detection limit of 7 ppmv was estimated (for 0.925-m-long HCF). In [29], the authors presented a HCF-based multigas Raman spectrometer. This setup achieved a 0.2 ppm detection limit for CH_4_ but required a high power laser source to be coupled into the small diameter fiber. Here, the detection limit of 500 ppbv was obtained for 1 second averaging, using 1.3-m-long HCF. Ambient methane detection was also demonstrated (to our knowledge for the first time using hollow-core fiber as a gas cell). We have also shown that Kagome-type fiber (which has core diameter >100 µm, comparable with some large diameter hollow core waveguides [30]) has significantly shorter gas exchange rates than PBG-type fibers (seconds vs. minutes). During the experiments, we have not seen any instabilities resulting from the gas flow through the fiber. 

In this work, Chirped Laser Dispersion Spectroscopy was used for gas detection. This technique relies on the molecular dispersion measurement, not absorption-based sensing [17]. The application of CLaDS resulted in an order of magnitude improvement of the detection limit comparing to our prior results obtained using similar optical layout and the absorption-based method (WMS) [19]. This is primarily because CLaDS is essentially a differential technique (we measure phase difference between multiple wavelengths, separated by modulation frequency Ω) which helps reduce the impact of unwanted periodic spectral features (such as optical fringes). The dispersion-based signal is also more immune to fluctuations of optical power in the system [17,22]; thus, it does not require the measured signal to be normalized with respect to the optical power that reaches the detector. The achieved performance (sensitivity) is comparable with previously demonstrated dispersion-based open-path systems [16]. However, the implementation of HCF as a gas cell provides several additional advantages, giving perspective for long optical path-lengths while maintaining small size and sample volume. This technology can be further extended to other molecular species and can be combined with less complex semiconductor sources such as interband cascade lasers (ICLs).
